# Heteroresistance of *Mycobacterium tuberculosis* in the Sputum Detected by Droplet Digital PCR

**DOI:** 10.3390/biology12040525

**Published:** 2023-03-30

**Authors:** Ye Win Aung, Kiatichai Faksri, Arunnee Sangka, Kanchana Tomanakan, Wises Namwat

**Affiliations:** 1 Department of Microbiology, Faculty of Medicine, Khon Kaen University, Khon Kaen 40002, Thailand; 2 Faculty of Associated Medical Science, Khon Kaen University, Khon Kaen 40002, Thailand; 3 Department of Medical Laboratory, Khon Kaen Hospital, Khon Kaen 40000, Thailand

**Keywords:** Droplet Digital PCR, heteroresistance, MDR-TB, *Mycobacterium tuberculosis*

## Abstract

**Simple Summary:**

Tuberculosis caused by *Mycobacterium tuberculosis* (MTB) is a major public health concern globally, and efforts to control and eliminate the disease continue to be a priority. Heteroresistance in MTB describes a bacterial population where distinct clones or subpopulations exhibit varying levels of antibiotic susceptibility. In this study, we employed Droplet Digital PCRs to investigate the heteroresistance of MTB in sputum samples in new TB cases. We targeted mutations in the *katG* and *rpoB* genes, which are associated with isoniazid and rifampicin resistance, respectively. This study found that in a total of 79 new TB cases, drug-resistant and drug-susceptible TB were detected in 11.4% and 88.6%, respectively. The prevalence of INH mono-resistant, RIF mono-resistant, and MDR-TB among total cases was 1.3%, 6.3%, and 3.8%, respectively. Heteroresistance in *katG*, *rpoB,* and both genes were found in 2.5%, 5%, and 2.5% of total cases, respectively. Our findings suggest that the detected mutations may have occurred spontaneously, as the patients had not yet received anti-TB treatment. The ddPCR’s ability to detect both mutant and wild-type strains in a population makes it a valuable tool for early diagnosis and management of drug-resistant TB. Improved methods such as ddPCR for detecting heteroresistance in MTB are crucial for global TB control and elimination.

**Abstract:**

Heteroresistance in MTB refers to the presence of distinct subpopulations of bacteria with varying levels of antibiotic susceptibility within a population. Multidrug-resistant and rifampicin-resistant TB are serious global health concerns. In this study, we aimed to determine the prevalence of heteroresistance in MTB from sputum samples of new TB cases using Droplet Digital PCR mutation detection assays for *katG* and *rpoB* genes, which are commonly associated with resistance to isoniazid and rifampicin, respectively. We found that out of 79 samples, 9 (11.4%) exhibited mutations in *katG* and *rpoB* genes. INH mono-resistant TB, RIF mono-resistant TB, and MDR-TB samples constituted 1.3%, 6.3%, and 3.8% of new TB cases, respectively. Heteroresistance in *katG*, *rpoB*, and both genes were found in 2.5%, 5%, and 2.5% of total cases, respectively. Our results suggest that these mutations may have arisen spontaneously, as the patients had not yet received anti-TB drugs. ddPCR is a valuable tool for the early detection and management of DR-TB, as it can detect both mutant and wild-type strains in a population, enabling the detection of heteroresistance and MDR-TB. Overall, our findings highlight the importance of early detection and management of DR-TB for effective TB control (in *katG, rpoB,* and *katG/rpoB).*

## 1. Introduction

Tuberculosis (TB) caused by *Mycobacterium tuberculosis* (MTB) is a significant public health concern with a global incidence of 10.6 million cases and 1.6 million deaths. Multidrug-resistant/rifampicin-resistant TB (MDR/RR-TB) affected 450,000 individuals in 2022. In Thailand, the incidence of TB was 143 per 100,000 population, and 11,000 deaths were reported [1]. The estimated number of MDR/RR-TB cases was 3.4 per 100,000 population in Thailand, making it one of the 30 high-TB-burdened countries [2].

Drug-resistant tuberculosis (DR-TB) includes mono-, multi-, and extensively drug-resistant TB. These categories are a challenge for TB control strategies worldwide. Mono-drug-resistant TB is resistant to only one first-line anti-TB drug. MDR-TB is resistant to the two most potent anti-TB drugs—both isoniazid (INH) and rifampin (RIF) [3]. Based on current estimates, the incidence of new cases of multidrug-resistant tuberculosis (MDR-TB) is nearly 500,000 per year on a global scale. The treatment of MDR-TB is expensive, prolonged, affects the daily life of sufferers, and can be life-threatening [4]. Mutations conferring resistance are seen in genes specific for each drug. For INH, the *katG* gene has such mutations. The most common is at codon 315 (S > T) and is found in 50–95% of INH-resistant clinical isolates [5], and in 82.95% [6], 56% [7], and 32% [8,9] of INH-resistant strains. The *katG* gene mutation at codon 315 (changed amino acid serine to threonine (AGC > ACC)) was found in 60% of isoniazid-resistant isolates using PCR-RFLP [10] and 94% of MDR-TB samples using sequencing [11]. For RIF, a single mutation in the *rpoB* gene is most commonly responsible for resistance. This mutation is at codon 531 (changed amino acid serine to leucine (TCG > TTG)), and is seen in 59.83% [6] and 46.3% [12] of RIF-resistant isolates. Additional mutations in the *rpoB* gene are S531L, D516V, and H526D, and were found in 41%, 21%, and 12% of MDR-TB samples, respectively, using the line probe assay [11].

Heteroresistance refers to a bacterial phenotype where subpopulations of cells show significant differences in antibiotic susceptibility, with the term used in the context of MTB to indicate the presence of varying susceptibility within the same clone (monoclonal heteroresistance) or several coexisting clones (polyclonal heteroresistance) [13]. Digital PCRs have been used to detect and quantify the heteroresistance of DR-TB in MTB H37Rv and XDR-TB mixed samples [14]. Droplet Digital PCR (ddPCR) is a multiplex PCR system that can simultaneously detect copies of mutant and wild-type alleles of target genes, including genes conferring drug-resistance in MTB. ddPCR can also detect very low concentrations of target DNA within small-volume samples without the need for a standard curve [15]. This system offers absolute quantification, superior partitioning, improved precision, and higher accuracy and sensitivity, even in the presence of PCR inhibitors [16]. ddPCR can be applied to detect heteroresistance for INH and RIF in the sputa of untreated TB patients. The aim of this study was to detect heteroresistance and drug resistance against MTB in sputum samples using ddPCRs.

## 2. Materials and Methods

### 2.1. Sputum Sample Collection

In this study, we utilized 79 residual sputum samples of new TB cases collected from Khon Kaen Hospital in Northeast Thailand. Our goal was to investigate the presence of heteroresistance associated with point mutations in two genes, *katG* and *rpoB*. Specifically, we aimed to detect mutations at codon 315 of the *katG* gene and codon 531 of the *rpoB* gene. To collect the sputum samples, we used wide-mouthed, screw-cap sputum containers that were then placed into plastic bags labeled with the biohazard symbol and *katG/rpoB.*

### 2.2. Sample Processing

Sputum samples were decontaminated using 1% N-acetyl L-cysteine sodium hydroxide digestion (1% NALC–NaOH). At least 2.5 mL of sputa was taken into 50 mL centrifuge tubes and equal amounts of NALC–NaOH were added. Then the tubes were tightly closed and vortexed for at least 20 s. They were incubated at 20–25 °C for 15 min. After that, phosphate buffer (0.067 mol/l, pH 6.8) was added up to the 45 mL mark on the tube. Tubes were centrifuged at 3000× *g* for 15 min. Following the sedimentation process, the supernatants were carefully and gradually discarded into a disinfected bottle, while the remaining sediment was redissolved with distilled water for subsequent procedures [17].

### 2.3. Nucleic Acid Extraction

After digestion and decontamination of the sputa using 1% NALC-NaOH, the bacterial pellets were dissolved in 1 mL of distilled water and transferred to 1.5 mL microcentrifuge tubes. After that, they were placed into the water bath (Memmert^®^, Schwabach, Germany) at 80 °C for 40 min to kill the bacteria [7]. They were centrifuged at 8000× *g* rpm for 1 min in a high-speed mini centrifuge (Biosan, Riga, Latvia). The supernatants were carefully removed. Genomic DNAs were extracted from the bacterial pellets remaining in the tubes using GF-1 Tissue DNA extraction kits (GF-TD-100, Vivantis, Selangor, Malaysia) according to the manufacturer’s instructions. To determine the concentration and purity of the extracted DNA samples, a Nanodrop 2000 c Spectrophotometer (Thermo Scientific^®^, Waltham, MA, USA) was utilized.

### 2.4. Detection of Heteroresistance Associated with Point Mutation in the katG Gene at Codon 315 and the rpoB Gene at Codon 531

In this method, we used 79 MTB-complex detected samples with ddPCRs using *mpt64* assay from our previous study [18]. We sought to detect the heteroresistance associated with mutation in the *katG* gene at codon 315 and the *rpoB* gene at codon 531 with ddPCRs using mutation detection assays for these two single-nucleotide polymorphisms (SNPs). When we amplified ddPCRs to detect the mutations, we used MTB H37Rv (reference strain) as the wild-type (WT) control and MDR-TB strain as the mutant (MT) control.

### 2.5. Designing Primers and Probes for ddPCR to Detect Mutation

#### 2.5.1. Mutation Detection Assay for *katG* S315T

The *katG* gene (2223 base pairs) is located between the c2156111 and c2153889 nucleotide sequence numbers in the complete genome sequence of *Mycobacterium tuberculosis* (H37Rv: GenBank accession number NC_000962.3). We targeted the most common mutation in this gene at codon 315, which causes an amino acid substitution—serine to threonine (AGC > ACC) [6,7]. The minimum required length of the target DNA sequence (61 base pairs on each side of the mutation at codon 315) was entered into the “digital PCR assay tab” at www.bio-rad.com (accessed on 14 September 2018). The website automatically provided details of mutation detection assays for *katG* S315T (Assay ID- dMDS684011294, Bio-Rad, Hercules, CA, USA). The PCR products were detected using a probe labeled “FAM” fluorophore (blue color) for the mutant allele or “HEX” fluorophore (green color) for the wild-type allele at the 5′ end, and an “Iowa Black” quencher for detecting non-template DNA at the 3′ end.

#### 2.5.2. Mutation Detection Assay for *rpoB* S531L

The gene of *rpoB* (3519 base pairs) is located between the sites 759,807 and 763,325, and contains nucleotide sequence numbers in the complete genome sequence of MTB (H37Rv: GenBank accession number NC_000962.3). The important mutation in this gene is at codon 531, which causes an amino acid substitution—serine to leucine (TCG > TTG) [12]. The minimum length of the target DNA sequence (61 base pairs on each side of mutation in the *rpoB* gene at codon 531) was entered into the “digital PCR assay tab” at www.bio-rad.com (accessed on 14 September 2018). The website automatically provided details of an assay (ID dMDS293228290, Bio-Rad, Hercules, CA, USA). The PCR products were detected using a probe labeled “FAM” fluorophore (blue color) for the mutant allele or “HEX” fluorophore (green color) for the wild-type allele at the 5′ end, and an “Iowa Black” quencher for receiving of non-template signals at the 3′ end.

### 2.6. ddPCR System and Its Reaction Conditions

To detect heteroresistance in TB patients, this study employed a ddPCR platform that consisted of the QX200 Droplet Generator, T100 Thermal Cycler, and QX200 Droplet Reader, all manufactured by Bio-Rad (Hercules, CA, USA). Each reaction was carried out with a total volume of 20 µL, containing 10 µL of 2× ddPCR supermix for probes (No dUTP), 1 µL of 20× FAM and HEX primers/probes for two mutation detection assays, 1 µL of Hind III (HF), 5 µL of nuclease-free water, and 3 µL of purified DNA samples. The ddPCR reaction mixtures and the generation oil for the probe (Bio-Rad, Hercules, CA, USA) were added separately into the eight wells of DG8 cartridges (Bio-Rad, Hercules, CA, USA), which were then loaded into the QX200 Droplet Generator for partitioning into nanoliter-sized droplets. The droplets were then transferred to a 96-well PCR plate (Bio-Rad, Hercules, CA, USA), and the plate was sealed using a heat-seal foil (Bio-Rad, Hercules, CA, USA) in a PX1TM PCR plate sealer (Bio-Rad, Hercules, CA, USA) at 180 °C for 5 s. The plate was subsequently loaded into the T100 Thermal Cycler, with the cycling conditions including an enzyme activation step at 95 °C for 10 min, 40 cycles, each comprising a denaturation step at 94 °C for 30 s, an annealing step at 50 °C for 1 min, and a final step at 98 °C for 10 min. The temperature ramp rate was set at 2 °C/s, while the best annealing temperature was found to be 50 °C for both mutation detection assays, which was selected from the optimized temperature gradient between 50–60 °C. All assays for each sample were carried out in duplicate. The confirmed MDR-TB strain acted as the mutant control, H37Rv as wild-type control, and nuclease-free water as non-template control in every reaction.

### 2.7. Limit of Detection (LOD)

In this study, we determined the limit of detection (LOD) of two mutation detection assays for *katG* S315T and *rpoB* S531L. The LOD was determined in duplicate using extracted and purified MDR-TB DNA as a positive control. The positive control DNA was diluted in nuclease-free water to create serial 10-fold dilutions from 1 ng/µL to 10^−6^ dilution or 1 fg/µL concentration of MDR-TB DNA. Our findings provide important insights into the sensitivity of these assays, which is crucial for accurate diagnosis and monitoring of MDR-TB infections (*katG/rpoB*).

### 2.8. Data Analyses

After completing thermal cycling, the 96-well PCR plate underwent analysis in a QX200 Droplet Reader. QuantaSoft^TM^ software, version 1.7.4 from Bio-Rad in Hercules, CA, USA, was used to interpret the FAM and HEX fluorescence intensities within the amplified droplets. This software enabled the measurement of the number of positive and negative droplets per fluorophore per sample, providing a comprehensive analysis of the PCR reactions [19]. The FAM fluorescence signal indicates the presence of the MT allele and HEX fluorescence signal indicates the WT allele in the droplets. Negative droplets containing non-template were shown below the threshold and positive droplets containing template DNA were above the threshold level. The QuantaSoft^TM^ software performed an automatic setup of the threshold level, with positive droplets registering above the established level and negative droplets registering below it, following the identification of qualified metrics as good data. The software was instrumental in interpreting and counting the number of positive and negative droplets per fluorophore in each sample, providing a comprehensive analysis of the PCR reaction. Moreover, the software utilized a specific formula to calculate the concentration (copies per microliter; CPM in a reaction mixture of ddPCR) using a unique algorithm, thereby enabling accurate determination of the target molecule’s concentration [19].

Concentration = −In (N_neg_/N)/V_droplet_

where In = inputting n, N_neg_ = number of negative droplets, N = total number of droplets, and Vdroplet = volume of droplets. The calculation of the DNA concentration in the sputum was used in the following formulas [18]:

Total copies = (CPM × V_reaction_/V_used DNA_) × V_used DNA_ × (C_total DNA_/C_used DNA_)

where CPM = CPM in the reaction mixture, V_reaction_ = volume of the total reaction, V_used DNA_ = volume of the used DNA, C_total DNA_ = concentration of the total DNA, and C_used DNA_ = concentration of the used DNA. To estimate the CPM in the sputum samples, the following equation was used:

CPM in sputum = Total copies in sputum/Volume of sputum

In this study, the k-nearest neighbor algorithm was applied to determine the threshold levels for differentiating positive and negative droplets based on fluorescence intensities in idol droplets [20]. The resulting data were analyzed using IBM SPSS Statistics software, version 20 (IBM Corp, Armonk, NY, USA), to investigate correlations between patients’ data and ddPCR results. Graphical charts were generated using GraphPad Prism 5 software (GraphPad, San Diego, CA, USA). These analytical tools were utilized to provide insights into the research findings and aid in the interpretation of the study outcomes.

## 3. Results

In our study, 79 MTB-complex detected samples were used and obtained from new TB cases with ddPCRs using the *mpt64* assay from a previous study [18]. The study revealed that the average age of the new TB cases was 53.6 ± 16.0 years. Among the total samples, 56 (70.9%) were male and 23 (29.1%) were female. This study found that 9 (11.4%) were DR-TB and 70 (88.6%) of the total 79 new TB cases were drug-susceptible TB. Among the nine DR-TB cases, one (11.1%), five (55.6%), and three (33.3%) were INH mono-resistant TB, RIF mono-resistant TB, and MDR-TB, respectively. Among the nine DR-TB cases, all INH-resistant samples (*n* = 4) included bacteria with the *katG* gene mutation at codon 315. This mutation was not present in any other samples, whether drug-resistant or -susceptible. Similarly, the *rpoB* gene mutation at codon 531 conferring RIF resistance was detected only in the eight samples that included RIF-resistant bacteria (Table 1).

In the present study, the FAM (MT) signals for the LOD of the mutation detection assay for *katG* S315T were observed to be 1 ng, 100 pg, 10 pg, 1 pg, 100 fg, 10 fg, and 1 fg of MDR-TB DNA, and were 224,851.6, 22,485.1, 2248.5, 224.8, 22.4, 2.2, and 0.2 CPM in the reaction mixture of ddPCRs, respectively. The LOD of the FAM (MT) signal for the *katG* S315T assay was found to be 10 fg (2.2 CPM) based on the comparison of copy numbers between the positive and negative controls (H37Rv-WT). The FAM and HEX signals of the LOD of MDR-TB and the negative control (10 fg of H37Rv DNA) for the *katG* S315T assay were 2.2/1.1 and 1.7/1.2 CPM in the ddPCR reaction mixtures, respectively. The range of FAM signals in the *katG* S315T assay for drug-susceptible TB and DR-TB was 0.0–1.6 CPM and 3.5–371 CPM in the ddPCR reaction mixtures, respectively. Establishing an appropriate cut-off value is crucial for the accurate identification of positive and negative samples in nucleic acid assays. It is important to take into account the LOD of the assay and the range of copy numbers in negative samples. This study determined that a cut-off value of 2.2/1.1 CPM for the FAM and HEX signals in the *katG* S315T assay, which corresponds to an LOD of 10 fg, was a reasonable threshold for identifying positive samples.

**Figure 1:**
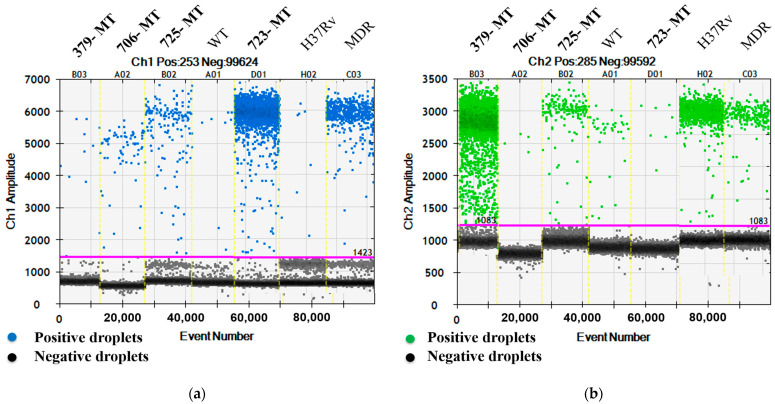
One D amplitude of (**a**) channel 1, FAM; (**b**) channel 2, HEX signals detecting MT and WT, respectively, of *katG* gene at codon 315 using mutation detection assay for *katG* S315T.

To detect *katG* gene mutations, the FAM (MT) and HEX (WT) signals in the four relevant samples (IDs 379, 706, 725, and 723) were 3.5/742, 5.8/0.33, 12.3/13.7, and 371/0.08 CPM in the reaction mixtures of ddPCRs, respectively. Among the four samples exhibiting the mutation of the *katG* gene at codon 315, sample number 706, was INH mono-resistant TB and the remaining samples (379, 725, and 723) were MDR-TB (Figure 1a,b).

The FAM (MT) signals for the LOD of the *rpoB* S531L assay were observed to be 1 ng, 100 pg, 10 pg, 1 pg, 100 fg, 10 fg, and 1 fg; and the positive controls were 371,391.3, 37,139.1, 3713.9, 371.3, 37.1, 3.7, and 0.3 CPM in the ddPCR reaction mixtures, respectively. The LOD of FAM (MT) signal for the *rpoB* S531L assay was discovered to be 10 fg (3.7 CPM) based on the comparison of copy numbers between the positive and negative controls (H37Rv-WT). The FAM and HEX signals of the LOD of MDR-TB and the negative control (10 fg of H37Rv DNA) for the *rpoB* S531L assay were 3.7/1.1 and 2.6/1.2 CPM in the reaction mixtures of the ddPCRs, respectively. The range of FAM signals in the *rpoB* S531L assay for drug-susceptible TB and DR-TB was 0.2–2.8 CPM and 4.7–205 CPM in the ddPCR reaction mixtures, respectively. The FAM and HEX signal cut-off values of 3.7/1.1 CPM in the *rpoB* S531L assay correspond to a limit of detection of 10 fg, and could be deemed an appropriate threshold for detecting positive samples (*rpoB).*

**Figure 2:**
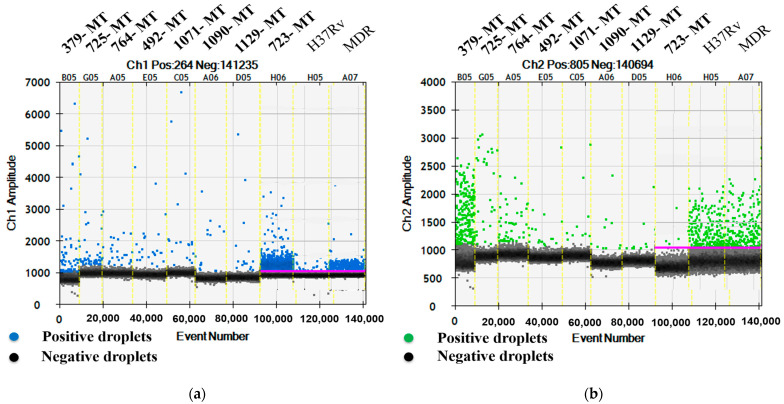
One D amplitude of (**a**) channel 1, FAM; (**b**) channel 2, HEX signals, detecting MT and WT, respectively, of *rpoB* gene at codon 531 using mutation detection assay for *rpoB* S531L.

To detect the *rpoB* gene mutation, the FAM (MT) and HEX (WT) signals in the eight relevant samples (IDs 379, 725, 764, 492, 1071, 1090, 1129, and 723) were 8.1/30.6, 5.6/3.2, 4.7/2.7, 5.8/1, 5.1/1.3, 10.2/1, 6.4/0.8, and 205/0.6 CPM in the ddPCR reaction mixtures, respectively. Among the eight samples exhibiting the mutation of the *rpoB* gene at codon 531, five (IDs 492, 764, 1071, 1090, and 1129) were RIF mono-resistant TB and the remaining samples (IDs 379, 723, and 725) were MDR-TB (Figure 2a,b).

The DNA concentration in sputum sample (ID-379) for the *katG* S315T assay was determined using the provided formulas:

Total copies (MT) = (CPM × V_reaction_/V_used DNA_) × V_used DNA_ × (C_total DNA_/C_used DNA_)
    = (3.5 CPM × 20 µL/3 µL) × 3 µL × (8232 ng/12.5 ng)
  = 46,099.2 copies               

CPM in sputum = Total copies in sputum/Volume of sputum
= 46,099.2 copies/2500 µL
= 18.44 CPM (MT)   

Total copies (WT) = (CPM × V_reaction_/V_used DNA_) × V_used DNA_ × (C_total DNA_/C_used DNA_)
    = (742 CPM × 20 µL/3 µL) × 3 µL × (8232 ng/12.5 ng)
= 9,773,030.4 copies             

CPM in sputum = Total copies in sputum/Volume of sputum
   = 9,773,030.4 copies/2500 µL
= 3909.21 CPM (WT)   

**Figure 3:**
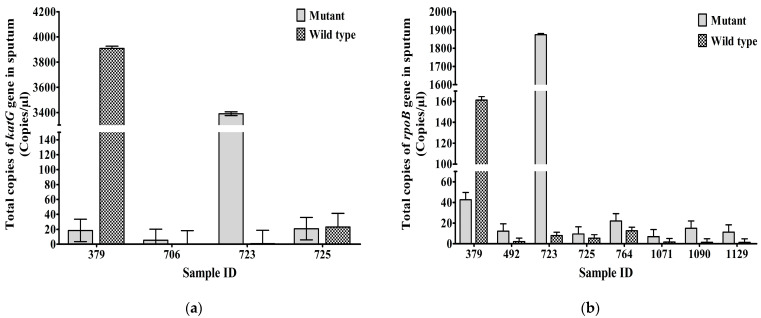
Total MT and WT copies (Mean± SEM) of (**a**) *katG* gene at codon 315 of INH-resistant TB, (**b**) *rpoB* gene at codon 531 of RIF-resistant TB, in sputum (CPM). Mean ± SEM is shown with error bars where SEM is computed by dividing standard deviation (SD) by the square root of the count (N).

Mean copy numbers of mutant/wild-type copies of *katG* gene at codon 315 in sputum samples 379, 706, 723, and 725 were 18.44/3909, 5.3/0.3, 3390/0.73, and 20.83/23.2 CPM, respectively (Figure 3a). The total number of MT/WT copies for the *rpoB* gene at codon 531 in sputum samples 379, 492, 723, 725, 764, 1071, 1090, and 1129 were 42.67/161.22, 12.32/2.12, 1873/7.86, 9.48/5.42, 22.11/12.7, 6.85/1.75, 15.08/1.48, and 11.35/1.42 CPM, respectively (Figure 3b).

**Figure 4:**
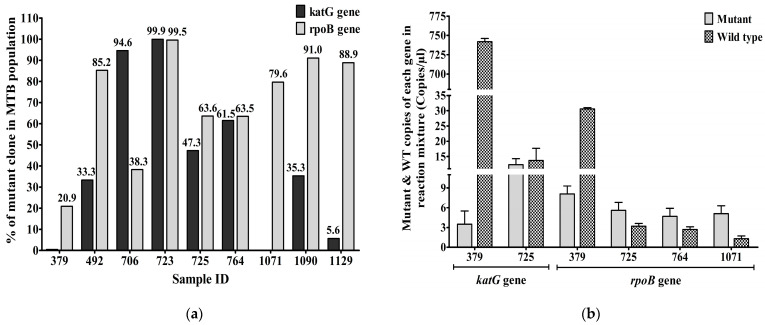
(**a**) The percentage of *katG* and *rpoB* mutant strains (Mean± SEM) in the MTB populations from 9 DR-TB cases based on sputum samples; (**b**) Mutant and WT copies (Mean± SEM) of *katG* and *rpoB* genes in DR-TB cases. Heteroresistance due to mutations in either or both the *katG* gene and the *rpoB* gene was detected in new TB cases. Mean ± SEM is shown with error bars, where SEM is computed by dividing standard deviation (SD) by the square root of the count (N).

The percentages of droplets containing the *katG* mutant strains at codon 315 in the MTB populations from samples 379, 492, 706, 723, 725, 764, 1071, 1090, and 1129 were 0.47%, 33.33%, 94.62%, 99.98%, 47.31%, 61.54%, 0%, 35.34%, and 5.66%, respectively. Samples 706 and 723 had >90% of droplets with the *katG* mutant strains. Percentages of droplets containing the *rpoB* mutant strains at codon 531 across the nine DR-TB cases were 20.93%, 85.29%, 38.3%, 99.58%, 63.64%, 63.51%, 79.69%, 91.07%, and 88.89%, respectively. Two samples, 723 and 1090, had >90% of droplets with the *rpoB* mutant strains (Figure 4a).

The mutant and wild-type alleles of *katG* genes at codon 315 of INH-resistant TB samples—379, 706, 725, and 723—were 3.5/742, 5.8/0.33, 12.3/13.7, and 371/0.08 CPM in the ddPCR reaction mixtures, respectively. Heteroresistance was detected in two cases, 379 and 725, based on the *katG* gene. The mutant and wild-type copies of the *rpoB* gene at codon 531 in RIF-resistant TB samples—379, 492, 723, 725, 764, 1071, 1090, and 1129—were 8.1/30.6, 5.8/1, 205/0.6, 5.6/3.2, 4.7/2.7, 5.1/1.3, 10.2/1, and 6.4/0.8 CPM in the reaction mixtures of ddPCRs, respectively. Therefore, four cases (379, 725, 764, and 1071) (50%) of RR-TB cases were heteroresistant according to the alleles of the *rpoB* gene, and two of these (379 and 725) were heteroresistant according to the alleles of both genes. Two (50%) of the four INH-resistant TB cases were also heteroresistant according to the alleles of the *katG* gene (Figure 4b).

## 4. Discussion

In the present study, DR-TB and drug-susceptible TB were detected in 11.4% and 88.6% of new TB cases, respectively. WHO reported in 2017 that the prevalence of MDR-TB was 4.7% globally and 2.5% among new TB cases in Thailand. MDR-TB was found in 81.6% of RR-TB cases globally [21]. In this study, MDR-TB, INH mono-resistant TB and RIF mono-resistant TB made up 3.8%, 1.3%, and 6.3% of the total of 79 new TB cases, respectively. Therefore, INH-resistant TB and RIF-resistant TB cases were 5.1% and 10.1% of new TB cases, respectively. The previous study showed that the average mutation of the *katG* gene is significantly higher in patients previously treated with INH (68.8%) compared to those without previous INH exposure (31.2%). These results suggest a potential association between INH exposure and increased *katG* gene mutation rates in TB patients [22]. The *rpoB* gene mutation was found in 85.2% of RR-TB and 8.9% of rifampicin-susceptible TB on average [23]. The present study found a 5.1% mutation rate in *katG* and 10.1% in *rpoB* genes prior to isoniazid and rifampicin treatment. We found that 75% of INH-resistant TB cases and 37.5% of RR-TB cases were MDR-TB using the ddPCR mutation detection assays. The prevalence of MDR/RR-TB was 3.5% of new TB cases and 18% of previously treated TB cases according to the *Global Tuberculosis Report 2018* [24]. A previous study showed that MDR-TB, INH mono-resistant TB, and RR-TB prevalence were 2.8%, 5%, and 3.4%, respectively [25]. Another study showed that the prevalence of INH mono-resistant TB was 5.1% in confirmed TB cases [26] and INH mono-resistant and RIF mono-resistant TB constituted 7.4% and 2% of AFB smear-positive samples [27].

The percentages of *katG* and *rpoB* mutant strains within a population of DR-TB were 0.47–100% and 20–100%, respectively. The percentage of mutant strains in heteroresistance of the *katG* gene was 0.5–50%, and the remaining INH-resistant TB was 95–100% of mutant strains within a population—and they were almost completely resistant to INH. The percentage of mutant strains in heteroresistance of the *rpoB* gene was 20–80%, and the remaining RR-TB was 85–100% of mutant strains within a population and they were almost completely resistant to RIF. A previous study showed that heteroresistance detection in the *katG* gene was 31% of INH-resistant TB cases [28]. The present study found that heteroresistance of MTB strains associated with INH, RIF, and both anti-TB drugs. Heteroresistance detection in *katG* and *rpoB* genes were 50% of INH-resistant TB and 50% of RIF-resistant TB cases. The heteroresistance (HR) may arise from a mixed infection, when resistant and susceptible strains infect a person at the same time, or while a single clone changes from a susceptible strain to resistant by undergoing genetic mutation under antibiotic pressure. In our study, the HR might possibly be either monoclone or heteroclone HR. Although there was no evidence to support the heteroclonal HR in Khon Kaen tuberculosis patients, the data that we collected support the possibility of monoclone HR (data not showed). Another study found that 8.5% and 14.2% of TB patients were heteroresistant to INH and RIF, respectively [29]. In this study, heteroresistance in *katG*, *rpoB,* and both genes were 2.5%, 5%, and 2.5% of new TB cases, respectively. However, the use of ddPCR is limited due to its high cost, lengthy turnaround time, and the need for skilled personnel.

## 5. Conclusions

We found that some samples contained INH/RIF-resistant clones in nearly 100% of MTB populations. This was because these patients had not yet started anti-TB treatment at the time of sample collection. Therefore, these non-treated patients could have acquired the resistant strain from other patients. The prevalence of MDR-TB in this study was higher than that listed in the 2018 WHO report for Thailand. Therefore, ddPCRs should be undertaken to surveil new cases on a regional basis for early detection of the transmission of TB, DR-TB, and MDR-TB, and should try to reduce the TB burden by giving effective anti-TB regimens. ddPCR is excellent for surveillance purposes because this system offers absolute quantification, superior partitioning, higher sensitivity, and improved accuracy for detection of resistance-conferring mutations. Moreover, ddPCRs can: a) detect both the mutant and wild-type strains simultaneously in a population, b) detect heteroresistance in MTB, c) may have important implications for the detection of MDR-TB, and d) could be a useful tool for the early diagnosis and management of DR-TB. The development and validation of more sensitive and accurate methods, such as ddPCR for detecting heteroresistance in MTB, may be a critical step in the global effort to control and eliminate tuberculosis, and may ultimately contribute to the surveillance of the drug-resistant pattern of TB.

## Figures and Tables

**Table 1:** Characteristics of DR-TB and drug-susceptible TB.

Characteristics	DR-TB *, *n* (%)	Drug-Susceptible TB, *n* (%)	Total, *n* (%)
Type of TB			
TB	0 (0.0%)	70 (100.0%)	70 (88.6%)
INH mono-resistant TB	1 (11.1%)	0 (0.0%)	1 (1.3%)
RIF mono-resistant TB	5 (55.6%)	0 (0.0%)	5 (6.3%)
MDR-TB	3 (33.3%)	0 (0.0%)	3 (3.8%)
*katG* at codon 315			
Detected	4 (44.4%)	0 (0.0%)	4 (5.1%)
Not detected	5 (55.6%)	70 (100.0%)	75 (94.9%)
*rpoB* at codon 531			
Detected	8 (88.9%)	0 (0.0%)	8 (10.1%)
Not detected	1 (11.1%)	70 (100.0%)	71 (89.9%)

* DR-TB was based on the results of ddPCRs using the two mutation detection assays.

## Data Availability

All data related to this study are presented in Table 1 and Figure 1, Figure 2, Figure 3 and Figure 4.
